# Hyperectatic Herniation of the Tympanic Membrane With Adenoid Vegetation

**DOI:** 10.7759/cureus.74651

**Published:** 2024-11-28

**Authors:** Jung Eun Shin, Minho Jang, JiAh Kim, Chang-Hee Kim

**Affiliations:** 1 Otolaryngology - Head and Neck Surgery, Konkuk University Medical Center, Seoul, KOR

**Keywords:** adenoid vegetation, eustachian tube dysfunction, hyperectatic herniation, otalgia, tympanic membrane

## Abstract

A 31-year-old woman presented with intermittent otalgia in the right ear, reporting severe pain during flights. The patient denied performing habitual Valsalva maneuvers. Otoendoscopic examination revealed hyperectatic herniation of the posterior portion of the right tympanic membrane (TM). Nasopharyngoscopy showed adenoid vegetation, and tympanometry demonstrated an abnormally high peak, indicating a thinned TM. The temporal bone CT was unremarkable, but a CT of the paranasal sinuses revealed a mild homogeneous lesion in the nasopharynx. The patient underwent adenoidectomy and right ventilation tube insertion, leading to significant improvement in TM herniation. This case suggests that hyperectatic herniation may occur due to impaired Eustachian tube function and recurrent otitis media, even in the absence of habitual Valsalva maneuvers. Unlike most previous reports associating TM bulging with Valsalva maneuvers, this case highlights other factors, such as barotrauma from flying, which may also contribute. Treatment options should be based on the severity of TM bulging, with observation or reduced Valsalva frequency in mild cases and ventilation tube insertion or other surgical intervention in severe cases. Further investigation into optimal management strategies for hyperectatic herniation of the TM is warranted, considering individual patient history and contributing factors.

## Introduction

The tympanic membrane (TM) forms the lateral wall of the middle ear cavity and the medial wall of the external auditory meatus. The horizontal and vertical diameters of the TM range from 8 to 9 mm and 8.5 to 10 mm, respectively, and the surface area of the TM is 85 mm² [1]. Electron microscopic studies demonstrated that the TM is a three-layered concave membrane composed of epidermis, lamina propria, and mucosa [2, 3]. The epidermal layer is a continuum of the epidermis of the external auditory meatus and consists of the four strata characteristic of skin (corneum, granulosum, spinosum, and basale) [1-11]. The lamina propria is composed of subepithelial and submucosal connective tissue layers with varying mixtures of collagen fibers and fine fibrils [5]. The outer radiate fibrous layer contains more fine fibrils, and the inner circular fibrous layer contains more collagenous fibers. Two dense fibrous layers provide the structural support for thin external auditory meatus skin laterally and the middle ear mucosa medially. Elastic fibers are rare in the pars tensa of the TM [7, 9, 10], and it was reported that the elasticity of the TM is 4.9 X 10-8 dynes/cm^2^, which is similar to that of rubber [6]. Thus, under normal conditions, outward bulging of the TM is extremely rare because the TM is less elastic and under tight tension.

Outward bulging of the TM has been reported as hernia, hyperectasis, or pulsation hernia in the previous studies [12-16], and positive pressure in the middle ear cavity due to repeated autoinflation has been suggested as a possible cause of this condition [16]. Although bulging of the TM is commonly observed in acute otitis media, otitis media with effusion, or middle ear tumors, ballooning herniation of a part of the TM is extremely rare [12].

In the present study, we report the case of a 31-year-old woman with hyperectatic herniation of the right TM with adenoid vegetation, treated by adenoidectomy and ventilation tube insertion.

## Case presentation

**Figure 1:**
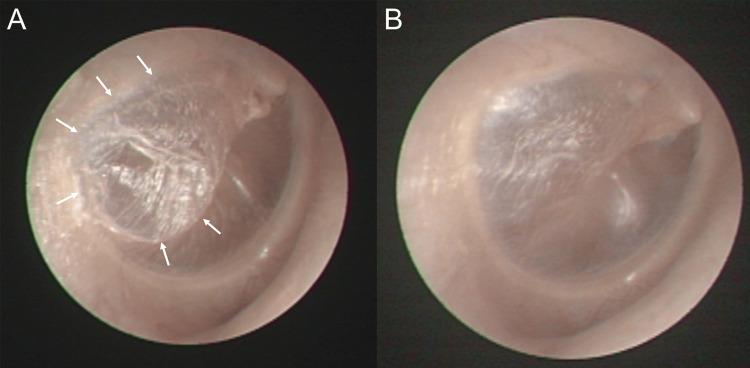
Otoendoscopic examination performed on the patient (A) At the first visit to our clinic, hyperectatic herniation was demonstrated in the posterior portion of the right tympanic membrane; (B) After extrusion of the ventilation tube, the improvement in hyperectatic herniation was observed.

A 31-year-old woman presented with intermittent otalgia on the right side in November 2018. The patient reported that she had been diagnosed with otitis media in the right side when she had upper respiratory tract infections since childhood. She experienced severe otalgia in the right ear every time she took a flight. Despite severe otalgia, she did not visit an ENT clinic to check the condition of her ear at those times. The patient denied performing habitual Valsalva maneuvers and did not have snoring or sleep apnea. Besides intermittent otalgia, she did not complain of other ear symptoms such as hearing loss, ear fullness, or tinnitus. The patient did not complain of rhinitis symptoms such as nasal obstruction, rhinorrhea, or sneezing. Otoendoscopic examination revealed hyperectatic herniation of the posterior portion of the right TM (Figure 1A) and a normal left TM.

**Figure 2:**
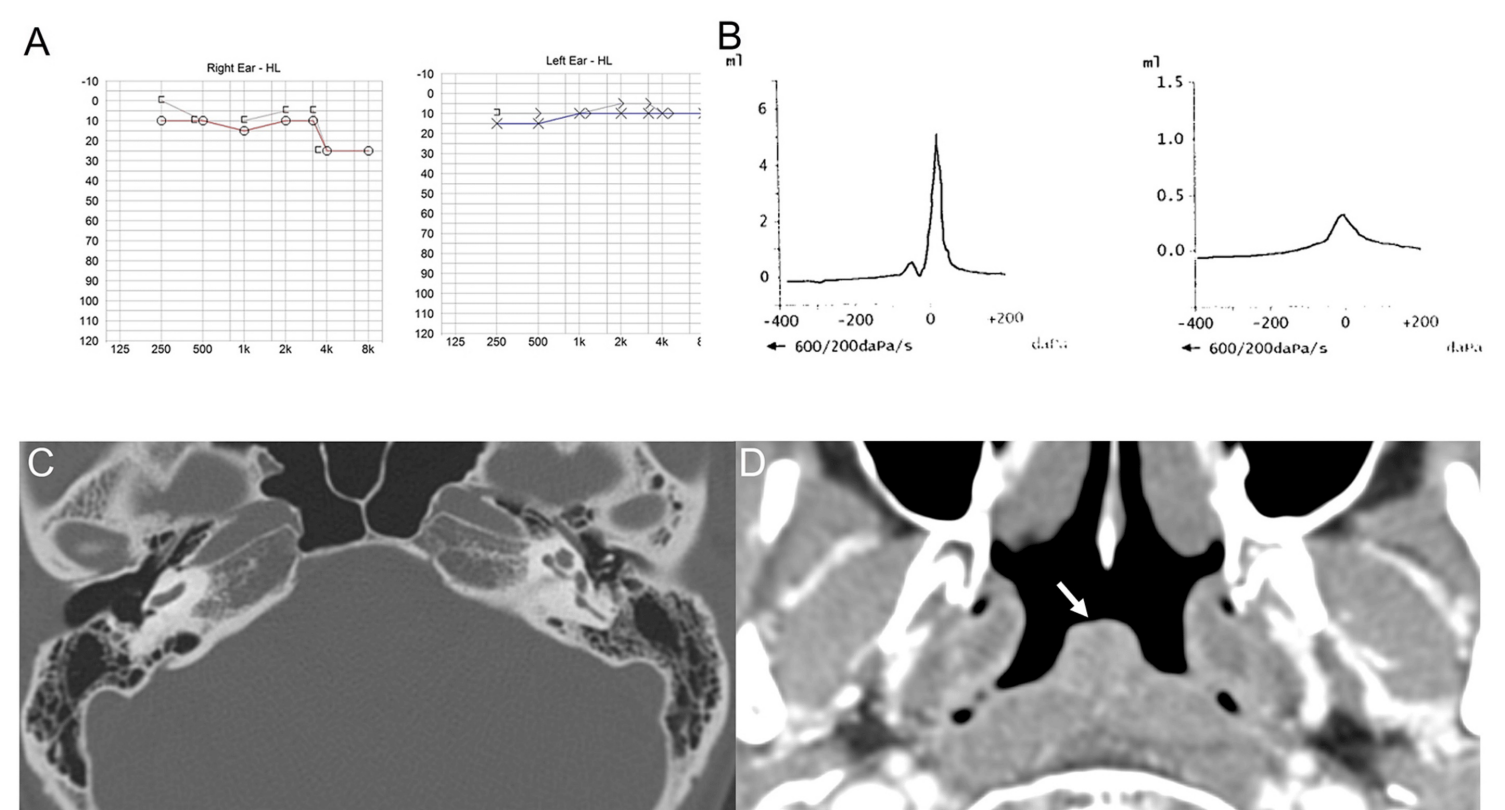
Audiometry and computed tomography (A) Pure tone audiometry revealed normal hearing thresholds on both sides; (B) Tympanometry demonstrated an AD Type in the right ear (left panel), indicating a thinned tympanic membrane, while tympanometry was A Type in the left ear (right panel); (C) Temporal bone computed tomography (CT) showed no abnormal findings on both sides; (D) Postcontrast paranasal sinuses CT showed a mildly enhancing, homogeneous, mass-like lesion in the mucosal area of the midline of the nasopharynx (white arrow).

Nasopharyngoscopic examination showed adenoid vegetation with adenoid size grading of Grade 2 in the posterior wall of the nasopharynx, and the nasopharyngeal end of the Eustachian tube was edematous. Pure tone audiometry showed normal hearing thresholds on both sides, except for mild high-frequency hearing loss in the right ear (Figure 2A). Tympanometry demonstrated that the height of the admittance tympanogram was increased in the right ear (Type AD) compared to the left ear, indicating a thinned TM on the right side (Figure 2B). Temporal bone computed tomography (CT) showed no abnormal findings in either the middle ear or mastoid cavities (Figure 2C), and paranasal sinuses CT showed a 1.2 x 1.6 cm mildly homogeneous enhancing lesion in the middle of the nasopharynx (Figure 2D).

Adenoidectomy with the right ear ventilation tube insertion was performed in February 2019. Myringotomy for tube insertion was carried out in the healthy, antero-inferior quadrant of the TM. The pathological diagnosis of the nasopharyngeal mass was chronic adenoiditis with lymphoid follicular hyperplasia. The ventilation tube extruded eight months after the procedure, and the patient did not experience otalgia during the eight months when the ventilation tube was in place. After the extrusion of the ventilation tube, the hyperectatic herniation of the right TM was much improved (Figure 1B). The patient scheduled a follow-up visit one year later, and upon returning after a year, the right TM findings remained unchanged compared to the previous year. We recommended that she avoid flying as much as possible, and she has not visited our clinic for any recurrence of otalgia four years since then.

## Discussion

The ‘pulsion hernia’ or ‘hyperectasis’ of the TM has been associated with autoinflation or habitual Valsalva maneuver in most patients of the previous reports [12-16]. High pressure is transmitted to the middle ear cavity through the Eustachian tube during the Valsalva maneuver, consequently applying pressure outward on the TM. Sadé suggested that hyperectasis of the TM is caused by a long-standing new steady state of the middle ear pressure above atmospheric pressure and described hyperectasis as a condition where either a small or large portion of the TM bulges above the physiological level [16]. Defect or thinning of the fibrous layer of the TM may precipitate herniation of the TM when the middle ear pressure becomes higher than atmospheric pressure [15]. In contrast to most patients in previous studies, our patient denied habitual Valsalva maneuvers or autoinflation. Considering that she had adenoid vegetation and experienced recurrent otitis media with effusion in the right ear, Eustachian tube function might be impaired on the right side [17-21]. Additionally, there is a possibility that a defect or thinning of the right TM may have been caused by recurrent inflammation. Because the patient reported experiencing severe pain in her right ear every time she flew, we speculate that repeated Valsalva maneuvers during flights due to poor equalization may be a cause of hyperectatic herniation of the TM in our patient. Sadé reported a long-term survey of 59 patients with hyperectasis of the TM and observed that the ballooned part of the TM was usually thin and scarred, and that hyperectasis was preceded by atelectasis, with both conditions alternating in many cases [16]. In Sadé's series of patients, nine had a history of acute secretory otitis media, four had a history of TM perforation, and four underwent tympanomastoidectomy due to cholesteatoma [16]. Lateral mastoid radiography demonstrated that hyperectasis, like atelectasis, is associated with poor pneumatization of the mastoid cavity, suggesting that a hyperectatic TM is attributed to a limited ability to buffer pressure changes [16]. Thus, as in our patient, hyperectatic herniation of the TM does not necessarily develop in association with habitual Valsalva maneuvers. However, because the Eustachian tube function test was not performed on our patient, the pathophysiology of TM hyperectatic herniation cannot be elucidated. It is recommended to perform the Eustachian tube function for the evaluation of the patients with hyperectatic herniation of TM.

There is controversy regarding the treatment of hyperectatic herniation of the TM because the current therapeutic approach is based on individual case reports. The choice of treatment modality generally depends on the severity of the TM bulging. Observation or a reduction in Valsalva maneuver frequency is recommended in mild cases [12-16]. Ventilation tube insertion can be considered to equalize middle ear pressure [12, 14]. In contrast, Fayad and House suggested that placing a ventilation tube might worsen TM bulging [13]. In extremely severe cases, surgical treatment with hernia excision and tympanoplasty is generally accepted [12, 14]. Since Eustachian tube dysfunction may be an underlying cause of hyperectatic herniation of the TM, balloon tuboplasty for obstructive Eustachian tube dysfunction and medical treatment with nasal steroids, topical decongestants, or antihistamines can be considered as combination therapy [12].

## Conclusions

Hyperectatic herniation of the TM refers to the outward bulging of the TM. A positive pressure in the middle ear cavity due to repeated autoinflation has been suggested as a possible cause of this condition. Defects or thinning of the fibrous layer of the TM may precipitate herniation of the TM when the middle ear pressure becomes higher than atmospheric pressure. Although hyperectatic herniation of the TM is mostly caused by habitual Valsalva maneuvers or autoinflation, this condition may occur in patients with a previous history of recurrent otitis media or barotrauma, even without habitual Valsalva maneuvers. Our patient was successfully treated with ventilation tube insertion and adenoidectomy.
